# Skin Development and Disease: A Molecular Perspective

**DOI:** 10.3390/cimb46080487

**Published:** 2024-07-30

**Authors:** Iasonas Dermitzakis, Despoina Chatzi, Stella Aikaterini Kyriakoudi, Nikolaos Evangelidis, Efstratios Vakirlis, Soultana Meditskou, Paschalis Theotokis, Maria Eleni Manthou

**Affiliations:** 1 Department of Histology-Embryology, School of Medicine, Aristotle University of Thessaloniki, 54124 Thessaloniki, Greece; iasonasd@auth.gr (I.D.); chatzidc@auth.gr (D.C.); kstellaai@auth.gr (S.A.K.); evangeln@auth.gr (N.E.); sefthym@auth.gr (S.M.); ptheotokis@auth.gr (P.T.); 2 First Department of Dermatology and Venereology, School of Medicine, Aristotle University of Thessaloniki, 54643 Thessaloniki, Greece; svakirlis@auth.gr

**Keywords:** skin, epidermis, appendages, hair follicle, sweat glands, development, embryogenesis, molecular cues, disease, cancer

## Abstract

Skin, the largest organ in the human body, is a crucial protective barrier that plays essential roles in thermoregulation, sensation, and immune defence. This complex organ undergoes intricate processes of development. Skin development initiates during the embryonic stage, orchestrated by molecular cues that control epidermal specification, commitment, stratification, terminal differentiation, and appendage growth. Key signalling pathways are integral in coordinating the development of the epidermis, hair follicles, and sweat glands. The complex interplay among these pathways is vital for the appropriate formation and functionality of the skin. Disruptions in multiple molecular pathways can give rise to a spectrum of skin diseases, from congenital skin disorders to cancers. By delving into the molecular mechanisms implicated in developmental processes, as well as in the pathogenesis of diseases, this narrative review aims to present a comprehensive understanding of these aspects. Such knowledge paves the way for developing innovative targeted therapies and personalised treatment approaches for various skin conditions.

## 1. Introduction

The skin, the largest organ and outermost layer of the human body, performs various functions [1]. These include maintaining hydrostatic pressure to prevent fluid loss, acting as a barrier against mechanical trauma and pathogen invasion, regulating body temperature, and facilitating sensory discrimination [2,3,4]. The skin comprises three primary layers: the epidermis, dermis, and hypodermis [5]. Within the epidermis, five distinctive layers are present [6]. The deepest one, known as the stratum basale or basal layer, consists of cuboidal or columnar cells [7]. Additionally, the basal layer hosts stem cells, allowing skin homeostasis, and serves as the anchorage site for migrating melanocytes and Merkel cells [8,9,10]. The following epidermal layer is the stratum spinosum, which is the thickest one. It consists of eight to ten bands of cells known as spines due to their polyhedral shape with centrally positioned nuclei. This region harbours Langerhans cells, which function as antigen-presenting cells [11]. Within the stratum spinosum, adjacent keratinocytes are bound tightly together through robust interactions facilitated by junctional complexes called desmosomes [12].

Diamond-shaped cells are organised in three to five bands and form another lamina above the spinous layer, known as the stratum granulosum. These cells contain keratohyalin granules, formations that enclose a protein called filaggrin [13]. Filaggrin undergoes decomposition to produce keratin bundles, while the resulting amino acids maintain the moisture of the cornified layer, ensuring the preservation of the skin’s barrier function [14,15]. Furthermore, lamellar granules filled with glycolipids promote cell cohesion and contribute to skin integrity within the stratum granulosum [16]. Subsequently, the stratum lucidum is only present in areas of thick skin, such as the palms and soles. It forms two to three layers of keratinocytes enclosing a product derived from the modification of keratohyalin known as eleidin [17]. The outermost layer of the epidermis, the stratum corneum, is composed of non-viable keratinocytes devoid of nuclei and organelles. These cells generate a stratified squamous epithelium with varying thickness to complete the skin barrier [18].

The basement membrane is a boundary between the basal layer, the innermost part of the epidermis, and the dermis. The dermis is a fibrous structure composed of elastic tissue and collagen, providing protection for the underlying skin structures [19]. It is anatomically divided into the superficial papillary layer and the deeper reticular layer. The last one is thicker and contains denser connective tissue compared to the papillary layer [20]. Within the dermis, various skin appendages such as sebaceous glands, hair follicles, sweat glands, sensory neurons, blood vessels, and muscles are located [21]. Afterwards, the hypodermis or subcutaneous fascia represents the deepest skin layer and insulates the body from temperature changes. Comprising mainly adipose and loose connective tissue, the hypodermis lacks collagen and elastic fibres. Nonetheless, it also shelters hair follicles, nerves, blood vessels, and muscles [22].

Skin ontogeny encompasses the intricate processes involved in the development and maturation of the skin during the embryonic and foetal stages of an organism’s life. These processes specifically entail the formation and differentiation of the various layers of the skin as well as appendageal growth. As evidenced in numerous tissues, the precise regulation of embryonic development by specific molecules is imperative in ensuring proper functionality [23,24,25]. Following suit with other biological systems, regulatory molecules may play a pivotal role in orchestrating the establishment of the skin layers [26]. The intricate molecular pathways and the genes involved in skin organogenesis have only recently begun to be elucidated, owing to the advancement of technological methods such as in vivo lineage tracing, immunofluorescence techniques, single-cell transcriptomics, and intravital imaging [27,28]. Despite these advancements, a comprehensive understanding of skin ontogeny remains challenging. Given the skin’s involvement in diverse pathological conditions, efforts must be directed towards this pursuit [29]. Disrupted embryogenesis, mutations, or dysregulations in genes encoding proteins essential for skin formation and resilience underlie many inherited skin diseases and various types of cancers, including epidermolysis bullosa, basal cell carcinoma, squamous cell carcinoma, and other pathological phenotypes [30,31,32,33].

This narrative review seeks to present molecular signals that coordinate the embryonic development of the skin. Moreover, this review aims to consolidate the existing literature to identify which molecular drivers are also implicated in the pathophysiological mechanisms underlying skin diseases. A comprehensive understanding of the theoretical foundations of skin disorders and associated cancers empowers scientists to leverage this knowledge in the advancement of more targeted therapeutic strategies.

## 2. Embryonic Development of the Skin

Epidermis development can be subdivided into the following stages: epidermal specification, commitment, stratification, terminal differentiation, and appendageal growth. Through these morphogenetic events, embryonic skin cells differentiate into specific cell types, forming the characteristic layers of the epidermis. Precise spatiotemporal regulation is necessary to create these intricate structures, mediated by various molecular cues and signalling cascades. Each stage of epidermal development is linked to the maturation of the underlying mesenchyme, emphasising the continuous communication between these tissues.

### 2.1. Specification

The epidermal specification is a developmental process during which early ectodermal cells undergo specific changes to acquire their epidermal fate. This process usually occurs in mouse embryos around embryonic day 8.5 (E8.5) [34]. Embryonic skin development begins immediately after gastrulation, a crucial stage in early embryonic development. Gastrulation involves the transformation of the embryo from a one-dimensional layer of cells into a multi-layered structure. This process includes the inward folding of the epiblast along the primitive streak, followed by the division and downward migration of epiblast cells. As a result, the three primary germ layers, namely ectoderm, mesoderm, and endoderm, are formed [35].

The ectoderm, one of these germ layers, is responsible for developing the nervous system and the skin epithelium [36,37]. Whether ectodermal cells will become neural or epidermal depends on the influence of Wnt, FGF, and BMP signalling after gastrulation [38,39,40]. Wnt signalling restricts the ectoderm’s response to FGFs. FGF signalling is crucial for neural induction; upon inhibition, it can lead the cells to acquire an epidermal fate [39,41]. The reduction in FGF signalling triggers the expression of BMPs, which inhibit neural development and commit the ectoderm to form the epidermis. This process shapes a single layer of versatile epithelial cells, known as the surface ectoderm, that covers the entire embryo. The surface ectoderm, comprising ectodermal progenitors expressing keratin 8/18, is then restricted to keratinocyte differentiation [27,34,42].

### 2.2. Commitment

Epidermal commitment is a crucial process that directs the primordial skin cells to adopt the fate of squamous stratified epithelial cells. During epidermal commitment, the surface ectoderm develops to become the embryonic basal layer, ultimately giving rise to all components of the future epidermis. This process is also essential for the formation of the basal membrane [34,43]. Additionally, a shift in keratin expression within the surface ectoderm occurs during epidermal commitment. Initially, a single layer of surface ectoderm cells expresses keratin 8/18, which is later replaced by keratin 5/14 before stratification begins. In mouse embryos, epidermal commitment occurs as early as E9.5 [44,45,46].

Keratins 5 and 14 are transcribed at high levels and are markers for cells with proliferative potential in the skin tissue [47]. The expression of keratins 5/14 is regulated by *p63*, a gene homologous to the tumour suppressor gene *p53*. Notably, *p63* is vital for epidermal commitment. It is predominantly active in the progenitor layers of the epidermis within the ectodermal surfaces of limb buds, branchial arches, and epidermal appendages. In these regions, a reciprocal signalling interaction occurs between the epidermis and the mesenchyme, influencing the morphogenetic organisation of the underlying mesoderm, which supplies essential growth factors for epidermal development [48,49]. Mice lacking the *p63* gene exhibit significant developmental defects in the limbs, craniofacial structures, and epithelial development, underscoring the critical role of *p63* in maintaining the cell populations of the surface ectoderm necessary for supporting keratinocyte development and morphogenesis [50].

**Figure 1:**
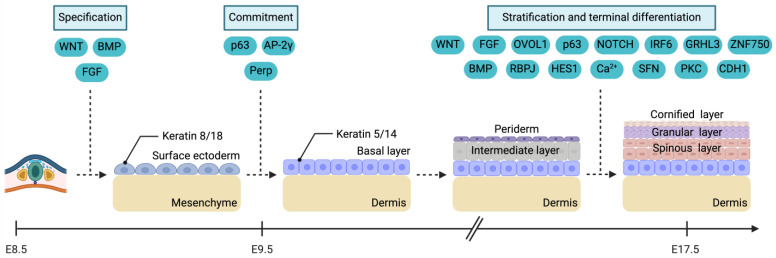
Molecular cues orchestrating epidermal specification, commitment, stratification, and terminal differentiation. AP-2γ: Activating protein 2γ. BMP: Bone morphogenetic protein. Ca^2+^: Calcium ion. CDH1: Cadherin 1. FGF: Fibroblast growth factor. GRHL3: Grainyhead-like transcription factor 3. HES1: Hes family bHLH transcription factor 1. IRF6: Interferon regulatory factor 6. Notch: Neurogenic locus Notch homolog protein. OVOL1: Ovo-like transcriptional repressor 1. Perp: P53 apoptosis effector related to PMP22. PKC: Protein kinase C. RBPJ: Recombination signal-binding protein for immunoglobulin kappa J region. SFN: Stratifin. ZNF750: Zinc finger protein 750. Created with BioRender.com.

ΔNp63 and TAp63, two isoforms of the p63 protein, are critical mediators in epidermal commitment, although the precise timing of their actions during this stage remains debated [51]. ΔNp63, a homolog of a nuclear protein specific to keratinocytes, interacts with a conserved motif within the enhancer of keratin 14, located upstream of the gene [52]. Studies on gene expression have shown that ΔNp63 can bind to a specific DNA sequence within the keratin 14 enhancer, leading to the transcription of the keratin 14 gene. This binding results in the shift in keratin expression needed for epidermal commitment and subsequent stratification. On the other hand, TAp63 regulates the expression of one or more AP-2 genes and induces AP-2γ expression in the embryonic epidermis of mouse embryos. AP-2γ is crucial for keratin 14 expression, as demonstrated by reduced keratin 14 levels upon the knockdown of AP-2γ expression in mice [51]. Furthermore, *p63* can directly activate the production of Perp, a tetraspan membrane protein component of desmosomes. Perp’s expression is essential for developing the basal layer by contributing to forming desmosomal adhesive complexes. Perp is also critical for maintaining the integrity of the basal layer and its adhesion to the basal membrane [53]. Ultimately, epidermal commitment culminates in forming the basal layer, originating from the surface ectoderm and serving as the precursor to all epidermal cells (Figure 1) [7].

### 2.3. Stratification and Terminal Differentiation

After epidermal commitment, a single protective layer of endodermal-like cells, known as periderm or “stratum germinativum,” covers the basal layer (Figure 1) [54,55]. Studies in mouse embryos using light and electron microscopy have shown that, by E9, the epidermis consists of a single cell layer. Periderm formation begins in the upper limb bud region around late E9, coinciding with the initiation of limb development. By E11, the periderm fully encases the surfaces of the limb buds, while in other regions, its formation remains incomplete until E12. Peridermal cells produce flat squamous protrusions that spread over neighbouring cells, detach from the basal lamina, migrate to the surface, and acquire a morphology resembling endothelial cells [56,57]. These findings indicate that the periderm is a protective barrier against regions with an actively developing basal epithelium [56]. In human embryos, the periderm emerges during the fourth to eighth week of gestation and is shed after the onset of stratification. Beneath the periderm, the basal layer gives rise to an “intermediate” layer of cells [37,58]. Although the presence of the periderm has been evidenced for a long period, its molecular identity has remained insufficient despite the application of scRNA-seq. Recently, a range of periderm markers, such as *Cldn6/23*, *Krt6/8/17/18/19*, *Grhl3*, *Sfn*, *Myh14*, *Paqr6*, *Tgfb2*, and *Sox9*, have been discovered [59]. The periderm goes through maturation and exhibits a distinct molecular signature; however, the precise functional role of molecular signals involved in the periderm ontogeny is yet to be completely elucidated.

Epidermal stratification is the process through which the embryonic epidermis develops into distinct layers, acquiring its characteristic stratified structure (Figure 1). Following epidermal commitment, the basal layer undergoes development and differentiation into specific cell types [27,60,61]. During stratification, basal layer cells proliferate and migrate upward. They form the transient intermediate layer beneath the periderm. This intermediate layer eventually divides and matures into the spinous layer. Terminal differentiation, where the spinous layer transitions into the granular and cornified layers, is typically observed around gestational weeks 22–24 in the interfollicular epidermis [60,62]. In mouse embryos, the completion of full epidermal stratification and differentiation is typically achieved by approximately E17.5 after the shedding of the periderm [56].

Two potential models exist regarding the initiation of stratification, with no clear preference for either. In the first model, the initiation of stratification occurs through the detachment of differentiated cells from the basement membrane after a decrease in integrin levels. Stem cells expressing high integrin levels remain undifferentiated and attached to the basement membrane [60,62]. The second model posits that, during stratification, at around weeks 8–11 of gestation, basal cells change their mitotic spindle orientation, undergoing asymmetric cell divisions with the spindle perpendicular to the basement membrane. This mechanism generates both committed suprabasal cells and undifferentiated basal cells [63,64,65]. Progression through asymmetric cell divisions relies on the transcription factor p63; the downregulation of p63 impedes intermediate layer development and disrupts stratification [64,66].

Cells within the intermediate layer lose their proliferative capacity, synthesising structural proteins and enzymes, including keratins and associated proteins [67]. Key keratins in the spinous layer, such as keratins 1 and 10, are induced by Notch signalling [47]. Notch signalling also regulates the proliferation and differentiation of basal cells, mainly through its effector Hes1 [68]. In the absence of RBPJ, a critical Notch-signalling mediator, spinous layers are absent in the mouse epidermis. Conversely, increased Notch1 signalling expands spinous layers. *Hes1*, a target of NICD/RBPJ, positively regulates spinous gene activation and the acquisition of spinous fate. However, the repression of basal genes is independent of *Hes1* [56,69]. The expression patterns of Notch receptors and ligands in different epidermal layers underscore the active role of Notch signalling in epidermal stratification [68]. Moreover, Wnt signalling has been implicated in epidermal stratification and terminal differentiation processes. Utilising a genetic mouse model that disrupts Wnt production in basal cells, it has been shown that Wnt production in the epidermis triggers a signalling cascade involving BMP and FGF in the underlying mesenchyme. This signalling cascade is crucial for activating the feedback regulation between the mesenchyme and the epidermis to control the formation of the spinous layer [70]. Additionally, *Ovol1*, *IRF6*, and *Sfn* regulate spinous layer maturation and are essential for inhibiting the proliferation of basal cells [71,72,73].

A single-cell transcriptome profiling study was conducted on the developing mouse epidermis at E14.5, E16.5, and P0, utilising both wild-type and *Grhl3^−/−^* mice [74]. The key finding of this study was the identification of the transcription factor GRHL3 as a crucial regulator of interfollicular epidermis differentiation, acting through the transcriptional activation of terminal differentiation genes. The process of murine epidermal differentiation was characterised as a single-step gradualistic process featuring a considerable population of transitional cells residing between the basal and spinous layers. CDH1 may also play a role in epidermal differentiation by facilitating altered adhesion properties that enable keratinocytes to undergo differentiation [59,75]. Regarding the epidermal master regulator, namely p63, it demonstrated binding to the *ZNF750* promoter and was essential for its activation. *ZNF750* restored differentiation in *p63*-deficient tissue, indicating its downstream role in the regulatory cascade initiated by p63. Importantly, *ZNF750* promotes terminal epidermal differentiation through the expression of late terminal differentiation genes without influencing the early ones [76].

Protein kinase C likely plays a role in converting spinous cells to granular cells. As cells differentiate to form the granular layer, they express filaggrin [15]. Extracellular Ca^2+^ concentrations closely control the terminal differentiation and maturation of the granular and cornified layers [77]. As cells progress toward the outermost layers of the stratum corneum, they undergo a process similar to apoptosis, becoming metabolically inactive. During this stage, they shed lipid bilayers and lose cytoplasmic organelles, including the nucleus. Between gestational weeks 24 and 26, the cornified layer begins to form [67,78]. It is important to note that another layer of the epidermis exists in adult skin, known as the stratum lucidum. This specific layer is only present in some regions of human skin, particularly in the fingertips, palms, and soles, located between the granular layer and the stratum corneum. The mechanisms governing the morphogenesis of the stratum lucidum remain incompletely understood [79].

### 2.4. Appendageal Growth

The appendageal growth of the skin involves the development of structures such as hair follicles, sweat glands, and sebaceous glands. This process is intricately regulated by molecular signalling pathways and cellular interactions to ensure the proper formation and maintenance of these essential skin components. In this review, we have examined both hair follicles and sweat glands because of their crucial roles in skin embryogenesis, physiology, and functionality. The literature concerning the molecular mechanisms that underlie the formation of sebaceous glands is scant.

#### 2.4.1. Hair Follicle (HF)

HF development relies on interactions between the epithelium and the underlying mesenchyme. In human embryos, the onset of HF development typically occurs around the 11th to 12th week of gestation. The progression of HF formation can be separated into eight discernible stages [80]. At stage 0, a basal layer of multipotent epithelial cells is established. At stage 1, localised condensations called placodes emerge in the embryonic epidermis in response to an inductive signal from the dermis [80,81]. Following this, stage 2 involves the downward elongation of the hair placode into the dermis, accompanied by the aggregation of dermal fibroblasts beneath the placode, forming dermal condensates (DCs). During stage 3, epidermal cells organise into a column structure, known as the hair peg, which comprises multiple cell layers. Simultaneously, the dermal fibroblasts in the dermal condensates give rise to the dermal papilla close to the hair peg [80]. In stage 4, the hair bulb thickens the peg, enclosing a segment of the elongated dermal papilla. At the same time, the inner root sheath begins to develop above the dermal papilla. Stage 5 sees the upward extension of the inner root sheath within the HF. In stage 6, the HF extends deep into the subcutis, with the inner root sheath forming the hair shaft and the formation of a sebaceous gland at the follicle’s apex [82,83]. Finally, in stage 7, the hair shaft enters the hair canal, and by stage 8, the hair shaft protrudes beyond the skin’s surface [84].

The development of HFs entails an exceptionally intricate and meticulously controlled process orchestrated by specific and numerous molecular signals. In mouse HF morphogenesis, the hair placodes form at E14.5 [85]. Before their emergence, epidermal *Wnt10b*, *Edar*, *Dkk4*, and *K17*, as well as dermal *Sox2* and *Sdc1*, are expressed [86]. In mouse embryos, the activation of β-catenin leads to increased epidermal Wnt activity [87]. The Wnt/β-catenin pathway is essential for initiating the first signal that triggers placode formation [88]. The deletion of β-catenin in the skin epidermis or overexpression of Dkk1, a Wnt signalling inhibitor, results in the absence of HFs [89,90]. Wnt signalling also contributes to the upregulation of Notch expression. Experiments involving loss-of-function or gain-of-function mutants of different Notch ligands and receptors revealed that this signalling pathway did not impact placode formation or the patterning of HFs. Notch assumed functional significance in the development of HFs during late embryogenesis, playing a crucial role in the differentiation of HF stem cells [91].

**Figure 2:**
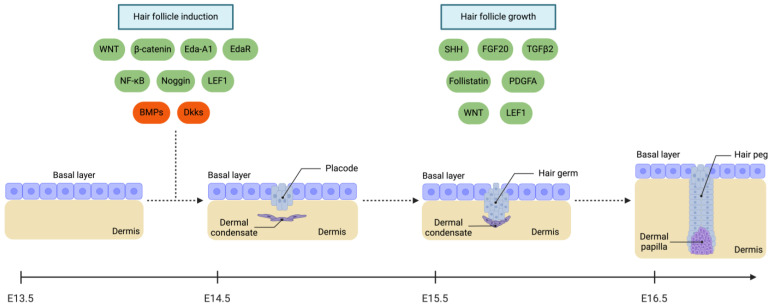
Molecular drivers regulating hair follicle fate specification and the initiation of its growth. Green-coloured cues represent the positive regulators, while red-coloured cues refer to the negative ones. BMPs: Bone morphogenetic proteins. Dkks: Dickkopf WNT signalling pathway inhibitors. Eda-A1: Ectodysplasin-A1. EdaR: Ectodysplasin A receptor. FGF20: Fibroblast growth factor 20. LEF1: Lymphoid enhancer binding factor 1. NF-κB: Nuclear factor kappa B. PDGFA: Platelet-derived growth factor A. SHH: Sonic Hedgehog. TGFβ2: Transforming growth factor beta 2. Created with BioRender.com.

Early in HF development, the Eda-A1/Edar/NF-κB signalling pathway is vital for inducing HF morphogenesis [92]. The Eda-A1/Edar/NF-κB pathway is necessary to maintain Wnt10a and Wnt10b levels in the hair placodes, ensuring the continued activity of Wnt/β-catenin signalling in the later stages of placode formation [87]. On the other hand, BMP signalling plays a role in inhibiting hair placode formation (Figure 2). It has been suggested that BMP signalling, while active in the region where the placode forms, diffuses into the interfollicular epidermis, suppressing follicular fate [93]. In both mouse and avian embryos, BMP2, BMP4, and BMP7 antagonists like noggin and follistatin maintain a delicate balance in follicle development by inhibiting BMP signalling [94,95,96,97,98]. Noggin expression by the underlying mesenchyme is essential for HF formation, partly through the induction of *Lef1* expression and the enhancement of the Wnt response [96,99]. Furthermore, follistatin plays a crucial role in the initial advancement of hair peg growth [86]. Specifically, the interaction between follistatin and activin is significant in HF development and cycling, potentially by controlling the expression of BMP2 [100].

Additionally, both Wnt and Shh signalling pathways regulate the specification of DCs. These pathways are further modulated by FGF20, secreted by hair placode cells [101,102]. Subsequently, the DC differentiates into the dermal papilla [102]. Interestingly, Shh was found to be non-essential for hair placode formation, while in the same line, a regular array of hair placodes still formed despite the absence of primary dermal condensations in *Fgf20* mutant mice [103,104]. Shh expression is vital for dermal papilla development, the transition from the hair placode to the hair peg, and the regulation of PDGFRA expression in the dermis [105,106,107]. During the down-growth phase of the HF, the dermal papilla expresses crucial signalling factors. Shh signalling, which is reliant on Wnt signalling, originates from the dermal papilla, and its importance in the morphogenesis of the developing HF is evident from its deletion, resulting in placodes and hair germs that are unable to penetrate the dermis and develop into various HF lineages [90,103].

HF invagination into the dermis is controlled by PDGF signalling. PDGFA is expressed in the HF placode, while PDGFRA is expressed in the dermal papilla [108]. Perturbations in PDGF signalling cause dermal papilla abnormalities, hindering the further development of the HF [105]. TGF signalling plays a pivotal role in HF down-growth and potentiates HF morphogenesis. Precisely, TGF-β2 transiently activates the transcription factor Snail and the MAPK pathway in the hair bud, promoting the proliferation of bud cells [109]. In TGF-β2-deficient mice, there is a significant reduction in the total number of HFs [110]. In the final stages of HF morphogenesis, which occur during the second trimester of pregnancy, a maturation process occurs, resulting in the development of seven layers of cells [85]. Throughout this maturation process, Wnt and BMP signalling pathways remain active, contributing to hair shaft differentiation [89,111,112]. FGF and Notch signalling pathways are also involved in the maturation of the HF [92]. In human embryos, a hair canal forms by the 21st week of pregnancy, followed shortly after that by the growth of the hair shaft. Mature HFs consist of a single layer of basal cells known as the outer root sheath, which express keratin-5 and keratin-14 and are in contact with the basement membrane [85,92].

#### 2.4.2. Sweat Glands

There are two types of sweat glands: eccrine and apocrine. Eccrine sweat glands, except for the lips and auditory canal, are widely distributed throughout the human body and comprise a single long and coiled sweat duct consisting of two layers of cells [113]. In human embryos, eccrine sweat gland development begins at around the 13th week of gestation and is completed by around the 24th week. In mouse embryos, eccrine sweat glands develop at E17.5 and reach maturity around two weeks after birth [114,115]. Apocrine glands, found only in hairy regions of the human body, start developing during the fifth month of gestation and originate from the upper part of the HF [116]. They have a brief functional period during the third trimester and remain inactive throughout the neonatal period. The exact origin of apocrine glands still needs to be fully understood [117].

During appendageal development, basal cells form buds, which are collections of progenitor cells. These buds invaginate into the dermis, eventually shaping HFs as well as apocrine and eccrine glands [117,118]. Eccrine sweat gland development involves the generation of a sweat duct and coiled gland originating from epidermal basal cells expressing keratin 5/14 [118]. A shared multipotent sweat gland cell precursor known as the “sweat bud progenitor” generates the sweat duct. The sweat gland duct comprises keratin 14-expressing basal progenitors and keratin 14/18-expressing suprabasal progenitors, which are multipotent in the initial stages of development [117,119]. As the sweat duct matures, these progenitors proliferate independently within their respective layers, eventually giving rise to the myoepithelial cells and luminal cells of the coiled gland [117]. By 22 weeks, myoepithelial and luminal cells in the secretory portion can be identified. Mesenchymal BMP stimulation initiates the sweat gland development process, while its inhibition favours HF generation [117].

In mouse embryos, the differentiation of the eccrine sweat gland placode from the HF placode requires the increased expression of epidermal *En1* and upregulation of the Wnt inhibitor Dkk4 [120]. Nevertheless, in humans, Wnt signalling, particularly its effector WNT10a, is crucial for eccrine gland induction and formation, as demonstrated by individuals with *WNT10a* mutations who displayed significant sweating abnormalities [121]. Wnt activity is detectable in the dermis of mouse embryos before the morphological signs of eccrine gland induction but decreases as gland germs form [122]. Epidermal Eda signalling, activated downstream of Wnt signalling, plays a vital role in complete sweat gland germ formation and upregulates the Shh signalling pathway in the early stages of development [121,123,124,125]. Moreover, the morphogenesis of the coiled sweat gland is a process potentially regulated by *Foxa1* and *Foxi1*, especially in late phases [126]. The exact role of these signalling cascades in sweat gland formation requires further elucidation.

### 2.5. Migration of Cells within the Epidermis

Various migration cells are located along the layers of the epidermis, originating from different anatomical regions of the embryo and migrating into the epidermis at different time points. These cells include melanocytes, Langerhans, and Merkel cells [10,127,128]. Regarding melanocytes, neural crest cells play a primary role in their formation [9]. Trunk melanocyte progenitors migrate dorsolaterally and express signalling pathways involved in melanogenesis, such as WNT, KIT, and EDNRB signalling, as well as distinct transcription factors like Mitf, FoxD3, Sox10, and Pax3 [129]. Upon reaching the epidermis, melanoblasts differentiate into melanocytes, producing melanin [130]. Melanocytes are present in the skin by week 12 of gestation in human embryos [131,132].

Langerhans cells are key components in the skin’s immune response, serving as antigen-presenting cells similar to dendritic cells [133]. These leukocytes are found in the spinous layer of the epidermis and originate from embryonic myeloid precursors in the foetal liver and yolk sac, sharing ontogeny with macrophages. Langerhans cells are derived through two pathways: c-myb-independent yolk sac macrophages and c-myb^+^ foetal liver monocytes, appearing in the foetal epidermis as early as week six of gestation [134,135,136,137,138]. Postnatally, Langerhans cells are self-renewable and not derived from bone marrow precursors. Their development and maintenance involve various TGF-β signalling pathways and transcription factors like Runx3 and Id2 [139,140,141]. The interaction between the CSF1R receptor and the keratinocyte-produced cytokine IL-34 is crucial for Langerhans cell differentiation [142]. Additional information on Langerhans cells can be found in relevant reviews [139,143].

Responsible for light touch sensation, Merkel cells are multimodal epidermal sensory cells that appear at around week 18 of gestation in human skin. Located in the basal layer and the lower epidermis at the dermal–epidermal junction, Merkel cells are closely associated with afferent neurons. Their origin is debatable, with some theories suggesting neural crest cells as the source and others proposing an epithelial/epidermal lineage based on the expression of epithelial keratins 8/18/20 [10,144]. Shh signalling is essential for Merkel cell specification in murine embryos, triggered by the Shh ligand produced in developing HFs. Merkel cell differentiation is strongly linked to HF morphogenesis [145]. The in-depth investigation of the ontogeny of migratory cells is not aligned with the scope of this review.

## 3. Molecular Basis of Congenital Skin Diseases and Cancer

Understanding the molecular mechanisms underlying congenital skin diseases and cancer has significantly advanced in recent years, providing crucial insights into the pathogenesis of these conditions. Researchers have uncovered key genetic mutations, signalling cascades, and cellular interactions that drive the development and progression of these disorders by unravelling the intricate molecular pathways affected. This section emphasises that the various molecular signals involved in embryonic development are also implicated in the pathophysiology of certain congenital skin diseases and types of cancer. Analysing these connections provides valuable insights into the molecular mechanisms underlying both skin development and the pathogenesis of skin cancers, such as basal cell and squamous cell carcinoma. Skin cancers often stem from disruptions in essential biological pathways that are also involved in normal skin development. Therefore, identifying these specific molecules can pave the way for developing new targeted therapies through laboratory-based interventions.

### 3.1. Congenital Skin Disorders

#### 3.1.1. Nevoid Basal Cell Carcinoma Syndrome (NBCCS)

NBCCS, also known as Gorlin syndrome, is an inherited disorder characterised by multiple developmental abnormalities and increased tumour predisposition. This condition follows an autosomal dominant inheritance pattern, with a noteworthy clinical characteristic being the presence of numerous jaw keratocysts, often accompanied by basal cell carcinoma. Predominant sites for cancer manifestation include the facial region, thorax, and dorsum [146]. Additional clinical features encompass fat herniation and ectopic calcification sites within the central nervous system, as well as a spectrum of skeletal, ophthalmological, genitourinary, and cardiovascular issues. Typically, the syndrome presents between adolescence and around 35 years of age, with childhood onset indicating a potentially more aggressive disease course [147,148]. While both NBCCS and basal cell carcinoma involve abnormalities in the Hedgehog pathway, the difference lies in the genetic predisposition and the multifocal nature of basal cell carcinomas in NBCCS patients compared to the sporadic occurrence in adults [149,150].

The gene associated with NBCCS is *PTCH1*, whose mutation is responsible for the syndrome. Its product, a transmembrane glycoprotein known as PATCHED1, acts as a tumour suppressor by regulating the cell cycle of epidermal cells [151]. Specifically, this glycoprotein serves as the receptor for SHH and, upon binding, inhibits the transcription of specific signalling molecules belonging to the Wnt and TGF-beta families [152]. Wnt signalling plays a critical role in epidermal development, renewal, and the formation of skin appendages. Additionally, it is involved in osteogenesis induction, the inhibition of adipose tissue formation, and the stimulation of fibroblasts. Consequently, the phenotypic manifestations of skeletal defects and fat herniation in NBCCS can be attributed to the disrupted physiological functions downstream of PTCH1/SHH signalling [153,154]. On the contrary, basal cell carcinoma could also arise from a variety of other genetic mutations. Therapeutic strategies for NBCCS include the surgical excision of affected areas, cryosurgery, carbon dioxide laser ablation, systemic drugs, and topical creams. More targeted therapies, such as gene therapy, remain an unexplored avenue in the management of this syndrome [155].

#### 3.1.2. Epidermolysis Bullosa (EB)

EB is a heterogeneous group of inherited dermatoses characterised by mucocutaneous fragility. Clinical manifestations include chronic injuries and blisters that occur spontaneously or following mechanical stress, as well as poor healing that may be accompanied by inflammation. Tissue separation occurs within the epidermis layers or between the epidermis and dermis. EB can be classified into four distinct subtypes, each with unique etiopathogenesis and symptoms [156].

Epidermolysis bullosa simplex (EBS) is associated with either autosomal dominant or recessive inheritance patterns. This condition arises from mutations in genes responsible for maintaining epidermal integrity, leading to the formation of blisters, itching, pigmentation irregularities, and potential discomfort [157]. The most commonly observed mutations occur in the *KRT5* and *KRT14* genes, which encode keratin 5 and keratin 14 proteins, respectively. These keratins are expressed prior to the epidermal stratification process, indicating the completion of epidermal commitment [44]. The expression of keratin 14 is regulated by the p63 protein, a member of the p53 protein family, and its absence hinders epithelial stratification, thereby impacting embryonic development and organs derived from the epithelial–mesenchymal lineage [52,158]. Following stratification and differentiation, keratin 5 and keratin 14 are also expressed in the basal membrane, where they interact to provide basal keratinocytes with flexibility and strength [159]. Reduced keratin expression compromises mechanical resilience, making cells more susceptible to proteolysis under increased mechanical stress [157]. Furthermore, cells with decreased keratin levels are more sensitive to heat, explaining the heightened blistering tendency, particularly in warm environmental conditions [160].

Mutations in the *DST*, *PLEC*, and *EXPH5* genes can be correlated with EBS. Normally, these genes are responsible for proper cytoskeletal organisation and lysosomal exocytosis [161,162,163]. Mutations in these genes have significant implications as they disrupt the structure of the keratin cytoskeleton in basal membrane cells, leading to the skin fragility and blistering characteristic of EBS [164]. Additionally, *KLHL24* and *CD151*, also known as tetraspanin 24, are genes implicated in EBS. Mutations in these genes indirectly affect the proteostasis of keratin. KLHL24 is part of a ubiquitination complex with K14 as its substrate. However, the mutations of *KLHL24* identified in EBS exhibit varied effects on the level of K14 expressed in patients [165,166,167]. Regarding the CD151 antigen, it plays a role in hemidesmosome formation, which are structures mediating integrin attachments between cells and the extracellular matrix by linking the basement membrane to the keratin filament network. Therefore, a defective CD151 antigen is associated with blistering and epidermal fragility in EBS [168].

Junctional epidermolysis bullosa (JEB) is an inherited autosomal recessive disorder characterised by skin blistering within the lamina lucida, a basement membrane component, and may also affect mucous membranes [31]. Several mutated genes are associated with JEB, three of which impact the proper function of laminin 332. Specifically, mutations in *LAMA3*, *LAMB3*, or *LAMC2* result in a defective laminin 332 protein, disrupting the function of anchoring filaments and the migration routes of keratinocytes during wound healing [164]. Laminin 332 is essential for skin stability through dermal–epidermal attachment [169]. Its involvement in epithelial function in organs beyond the skin contributes to systemic symptoms in severe JEB cases [170]. Integrins, particularly *ITGA6* and *ITGB4*, are also implicated in JEB. These genes encode integrin α6β4, a transmembrane receptor involved in hemidesmosome formation, mediating cell–matrix adhesion [171]. *ITGA6* is a molecular marker on neural crest cells’ skin derivatives during embryonic development, while *ITGB4* mediates epithelial–mesenchymal transition [172,173]. Mutations in either gene lead to a phenotype characterised by skin blisters due to the disrupted adhesion between the epidermis and dermis, resulting in loss of skin integrity and additional clinical features such as pyloric atresia from gastrointestinal mucosal dysfunction [174].

Another gene encoding integrins that is correlated with JEB is *ITGA3*. Specifically, *ITGA3* encodes the integrin α3 subunit and participates in the epithelial–mesenchymal transition process [175]. Aside from skin fragility, mutations in this gene result in nephrotic syndrome and interstitial lung disease, underlining the importance of integrin α3β1 for the proper function of keratinocytes, podocytes, and alveolar epithelial cells [176,177]. Mutations in *COL17A1* are also implicated in the pathogenesis of JEB, leading to the production of abnormal collagen XVII, a crucial component of hemidesmosomes that promotes stable binding between basal cells and the basement membrane [178]. Moreover, collagen XVII is a vital niche protein in the epidermis and HFs that maintains epidermal and melanocyte stem cells [179,180]. *COL17A1* expression in the interfollicular epidermis stem cell niches promotes keratinocyte differentiation [181]. Consequently, mutations in *COL17A1* can result in increased skin fragility, skin thinning, alterations in skin pigmentation, hair loss, and an elevated risk of cancer development [182,183].

Dystrophic epidermolysis bullosa (DEB), also known as recessive DEB, is a condition that causes fragility detected beneath the epidermis. Specifically, blistering occurs below the lamina densa level. The lamina densa, along with the lamina lucida, forms the basal lamina and lies on the dermal connective tissue, with its primary component being type IV collagen [184]. DEB results from mutations in the *COL7A1* gene, which typically produces type VII collagen necessary for maintaining the skin’s integrity and homeostasis [185]. Type VII collagen plays a significant role in anchoring fibrils, essential complexes for attaching the lamina densa to the upper papillary layer of the dermis [186,187]. Thus, mutations in *COL7A1* cause blistering and scarring. DEB also disrupts junctions in deeper skin layers, leading to mucosal involvement, including blistering in the oesophagus, anal canal, oral cavity, and eyes [188,189,190]. Type VII collagen is thought to influence the development of epidermal tumours, so patients with DEB may be at a higher risk of developing SCC [187].

A rare subtype of EB constitutes Kindler EB or Kindler Syndrome (KS) following an autosomal recessive inheritance pattern. Clinical manifestations encompass skin fragility and blister formation on the extremities from birth [191]. Other phenotypic characteristics include generalised cutaneous thinning, mottled skin pigmentation, sunlight sensitivity, widespread skin thickening on the palms and soles, pseudosyndactyly, and mucosal lesions [192]. KS is caused by a mutation in the *FERMT1* gene, producing a defective kindlin-1 protein [193]. During embryonic development, *FERMT1* appears to mediate epithelial–mesenchymal transition and plays a role in the adhesion, movement, and migration of epithelial cells [194]. This is accomplished by enhancing the affinity of integrins for their ligands, a phenomenon referred to as integrin activation. Subsequent integrin-mediated signalling pathways facilitate cell adhesion, migration, proliferation, and eventual differentiation [195]. In *FERMT1* mutants, faulty kindlin-1 leads to aberrant integrin activation, amplifying mechanical stress within keratinocytes upon skin damage. This provokes an increased release of cytokines and growth factors, stimulating dermal fibroblasts to produce excessive extracellular matrix, resulting in scarring and fibrosis [196,197]. The loss of kindlin-1 is associated with decreased TGFβ1 and increased Wnt/β-catenin signalling, affecting melanocyte homeostasis and contributing to observed poikiloderma in KS [198]. Furthermore, the disruption of skin homeostasis leads to epidermal atrophy in KS due to the sustained activation of stem cells through TGFβ1 and Wnt/β-catenin signals, culminating in stem cell depletion [195,199]. A comprehensive overview of the EB-related genes is provided in Table 1.

When considering the therapeutic approaches available for patients with different types of EB, apart from symptomatic treatment, a range of methods involving micromolecules, cell injections, and gene editing are employed [156]. Initially, gene therapy has shown promising outcomes in specific JEB and DEB cases, albeit largely limited to in vitro experimentation and early-stage clinical trials. In this therapeutic strategy, fibroblasts or keratinocytes are isolated from skin biopsies, genetically modified with the functional cDNA strand of the mutated gene, and subsequently reintroduced into the skin through grafting procedures [206]. Gene therapy was first administered to a patient diagnosed with JEB due to a mutation in the *LAMB3* gene, while clinical trials are investigating its efficacy in EB cases caused by mutations in *COL17A* [207,208]. The potential use of gene therapy in targeting mutations in *COL7A1* for DEB cases is also under evaluation [209].

An alternative treatment option for EB involves the intradermal injection of fibroblasts, leading to improved adhesion between the epidermis and dermis in DEB patients despite adverse events such as excessive pain [210]. Additionally, mesenchymal stromal cells derived from bone marrow have been administered intravenously to some children with DEB, resulting in overall health enhancement, even though collagen VII levels remained suboptimal [211]. The direct infusion of collagen VII has been explored as a potential treatment for DEB, yet its effectiveness requires further clarification [212]. Gene editing presents another avenue for EB therapy, facilitated by the development of designer endonucleases such as TALEN or CRISPR/Cas9, aimed at correcting mutations in keratinocytes associated with EB [213,214,215].

#### 3.1.3. Hypohidrotic Ectodermal Dysplasia (HED)

HED is characterised by sparse hair on the scalp and body (hypotrichosis), reduced sweating ability (hypohidrosis), and the congenital absence of teeth (hypodontia). Its inheritance may follow X-linked recessive, autosomal dominant, or autosomal recessive patterns [216]. X-linked HED is attributed to mutations in the *EDA* gene, typically responsible for ectodysplasin production. Ectodysplasin is a protein belonging to the TNF family and is essential for proper placode development [202]. Specifically, the EDA protein serves as a signalling molecule during embryogenesis, facilitating the interaction between the ectoderm and mesoderm, thereby orchestrating placode formation. These placodes eventually give rise to various ectodermal structures such as HFs, sweat glands, and teeth. The mutated EDA protein leads to multiple ectodermal deficiencies, accounting for the observed phenotype [217]. In some instances, X-linked HED may be associated with immunodeficiency, which arises from mutations in the gene encoding the NF-κB essential modulator, which is crucial for the induction of pro-inflammatory genes [203,218].

Mutations in *WNT10A*, *EDAR*, or *EDARADD* genes cause autosomal recessive and dominant HED (Table 1) [204]. Precisely, alterations in *WNT10A* disrupt the normal activity of the β-catenin pathway, which is crucial for the proliferation of epithelial progenitors. Additionally, *WNT10A* influences the specification process in certain epithelial regions through the transcription factor KLF4. Consequently, its mutation hampers the expression of specialised keratins in epithelial cells, resulting in abnormal skin structure and integrity [219]. The normal *WNT10A* gene is also involved in proper odontogenesis and the embryonic development of HFs, with modifications in its sequence explaining the hypotrichosis and hypodontia seen in HED [220]. On the other hand, *EDAR* and *EDARADD* genes are part of the Eda pathway, which is essential for forming structures derived from the embryonic ectoderm such as teeth, HFs, and sweat glands [202].

The treatment of HED primarily focuses on symptom management. Recently, a promising approach involved carbon dioxide laser therapy and appropriate ointments containing triamcinolone, lidocaine, and prilocaine, yielding positive outcomes [221,222,223]. For patients with X-linked HED, attempts have been made to replace the defective EDA1 protein postnatally with EDI200. This homologue can bind effectively to the EDAR receptor, promoting proper ectodermal development [224]. However, initial trials were unsuccessful in achieving sweat gland development [225]. Alternative strategies include the intra-amniotic administration of the EDA1 homologue to influence sweat gland embryogenesis. Some X-linked HED cases treated with this method have shown promising results, leading to ongoing clinical trials and new perspectives in the field [226].

### 3.2. Skin Cancer

#### 3.2.1. Basal Cell Carcinoma (BCC)

BCC is the most common malignancy among individuals with light skin colour, accounting for approximately two-thirds of all skin cancers in light-skinned people [227]. BCC’s molecular underpinnings are complex, involving a combination of inherited genetic predisposition and spontaneous somatic mutations. The inherited predisposition includes single nucleotide polymorphisms and hereditary conditions, while somatic mutations are usually an initiating factor that triggers tumour development [228]. BCCs constitute the majority of keratinocyte carcinomas and typically arise in sun-exposed areas of the skin, such as the head, neck, thorax, and lower limbs. In the context of sun-induced damage, oxidative stress serves as the connecting factor between sun exposure and molecular pathway dysregulation [229]. These tumours exhibit a slow growth pattern and rarely metastasise to other body parts. A characteristic feature of BCC is the formation of basaloid cell islands within the epidermis, some of which have the potential to invade the dermis [230,231]. Clinical presentations of BCC are diverse, with the carcinoma manifesting nodular, pigmented, superficial, or sclerosing patterns [232]. BCC is believed to originate from HFs due to the histological and biochemical characteristics shared between the two. Bulge stem cells appear to be BCC’s primary cellular source when it is triggered by ionising radiation [233,234].

In the molecular pathogenesis of BCC, the HH signalling pathway has a central role. Its primary function is to regulate the skin’s stem cell population and contribute to forming sebaceous glands and HFs [235]. Additionally, components such as the transmembrane receptors PTCH1 and PTCH2, SMO, GPCR, GLI1, GLI2, and GLI3 are part of the HH signalling cascade, interacting with the primary cilium [236]. The primary cilium is a microtubule-based cellular protrusion that senses extracellular signals [237]. The activation of the HH pathway occurs when HH molecules bind to PTCH1. In the absence of HH ligands, PTCH1 inhibits SMO, which is localised at the base of the primary cilium, thereby blocking further signalling. Upon the detection of external signals by the primary cilium, HH ligands bind to PTCH1, relieving the inhibition and allowing SMO to translocate to the ciliary tip [238]. In conjunction with other signalling molecules like SUFU, SMO activates the GLI transcription factors, which translocate to the nucleus and promote the expression of genes involved in cellular homeostasis, regeneration, and angiogenesis [239,240].

In most cases of BCCs exceeding 90%, loss-of-function mutations have been identified in the HH signalling cascade, predominantly involving the *PTCH1* gene. The disruption of this molecular pathway results in the dysregulation of the cell cycle through the impairment of SMO inhibition. This leads to the continuous activation of HH signalling and epidermal cell hyperproliferation, thus contributing to BCC development [240,241,242]. Rarely are genetic alterations also observed in the *SMO* gene, leading to the sustained activation of its transcriptional product. Additionally, loss-of-function mutations in the *SUFU* gene and the *PTCH2* gene have been reported in some cases. These alterations in critical protein molecules typically drive tumorigenesis by pathologically increasing GLI protein transcription. The excessive transcription of GLI proteins triggers uncontrolled proliferation of their target cells, furthering BCC progression [243,244]. Another common mutation in BCC development is the inactivation of the *TP53* gene, which typically acts as a tumour suppressor, promoting cell apoptosis [245,246]. Consequently, epidermal cells exhibit excessive proliferation tendencies, fostering the accumulation of additional mutations that drive tumorigenesis and culminate in BCC formation [247].

In addition to the direct role of the mutated *TP53* in BCC by disrupting the cell cycle, alternative mechanisms have been identified. In specific mouse models displaying BCC, the loss of *TP53* gene function led to the upregulation of SMO, thereby enhancing the HH molecular cascade, as previously indicated [234]. Similarly, the downstream effectors of the HH cascade, such as the MYCN/FBXW7 signalling pathway, are implicated in BCC pathogenesis [248]. Particularly, the MYC family of transcription factors, including MYCN, govern cell growth, proliferation, and the programmed cell death of keratinocytes in the epidermal layers. Thus, missense mutations in the *MYCN* gene could elucidate its role in BCC development, attributed to increased cell proliferation rates and reduced apoptosis [243].

Apart from the mutations previously mentioned in particular BCC cases, genetic alterations in the Notch signalling pathway have also been observed. This molecular cascade downregulates keratinocyte proliferation and is induced by the tumour suppressor protein p53 [249]. In individuals with BCC, Notch signalling is suppressed, enabling continuous keratinocyte proliferation, ultimately leading to tumour formation [250]. Moreover, Notch signalling governs the adhesion between keratinocytes and facilitates the differentiation of epidermal cells into spinous cells [251,252]. Another intriguing molecular pathway implicated in the pathogenesis of this keratinocyte carcinoma is the Hippo–YAP/TAZ pathway, which is integral to skin morphogenesis [253]. It specifically activates epidermal stem cells, contributing to the development of HFs, interfollicular epidermis, and sebaceous glands essential for skin homeostasis [254]. This pathway is upregulated, leading to the excessive proliferation of epidermal-derived cells, a key factor in BCC development [255,256]. New cases of BCC also involve mutations in gene promoters, with *TERT* and *DPH3* being among the most significant [257,258]. While surgical excision remains BCC’s primary treatment modality, radiotherapy and systemic therapies can also be beneficial. Nevertheless, exploring more advanced therapeutic strategies targeting the pertinent genes or proteins is essential for future advancements in BCC management [259]. A summary of the BCC-implicated genes is provided in Table 2.

#### 3.2.2. Squamous Cell Carcinoma (SCC)

SCC ranks among the most common human cancers not related to melanoma and is characterised by highly invasive behaviour with elevated mortality rates [271]. Clinically, it typically presents as red patches, hyperkeratosis, and irregular vessels, while more severe cases may exhibit ulcers, papillomas, and papules [272]. Environmental factors implicated in SCC include ultraviolet radiation, smoking, alcohol consumption, and infectious agents [273,274,275]. SCC often shows signs of squamous differentiation, suggesting a possible origin from cells, such as those in the interfollicular epidermis, that typically undergo squamous differentiation [276]. There is a debate regarding whether the initial genetic alteration occurs in stem cells with prolonged lifespans or in differentiated epidermal cells [276,277].

The etiopathology of this keratinocyte carcinoma subtype typically involves loss-of-function mutations in tumour suppressor genes, particularly in *TP53* and *CDKN2A* (Table 2) [260]. As mentioned above, P53 plays a crucial role in modulating the cell cycle [261]. On the other hand, CDKN2A functions by inhibiting cyclin-dependent kinases that regulate the activity of p105-Rb, a tumour suppressor protein encoded by the RB1 gene. In SCC, the suppression of p105-Rb function results in uncontrolled cell proliferation [262]. Additional mutations affecting the cell cycle process involve *CCND1*, which encodes for cyclin D1 and *MYC*. These mutations lead to hyperproliferation, characterising SCC [263]. Tumorigenesis resulting from the above mutations is typically not associated with infectious agents, such as exposure to the HPV virus, which has been implicated in some cases of SCC [278].

Furthermore, gain-of-function mutations are commonly observed in SCC. Specifically, the most frequently mutated genes include *FGFR1*, *FGFR2*, *FGFR3*, and *EGFR*, which encode growth factor receptors crucial for skin embryonic development [265,266]. The FGF signalling pathway facilitates mesodermal formation and maintains a balanced interaction between the mesenchyme and the epidermis, while *EGFR* is essential for keratinocyte proliferation. Mutations in these genes lead to excessive cell proliferation and reduced apoptosis, which are features of SCC [279,280]. Downstream of these receptors, the RAS and PI3K signalling pathways are impacted indirectly through altered interactions with their receptors and directly via de novo mutations in their sequences, resulting in reduced cell apoptosis that contributes to tumorigenesis in SCC [281,282]. Another gene implicated in SCC pathogenesis is *TP63*, which regulates the transition from simple to stratified epithelium and supports the maintenance of the skin SC population [48]. *TP63* is vital for squamous cell metaplasia, and its missense mutations are involved in SCC development [283].

Aside from *TP63*, *SOX2* is also implicated in squamous cell fate determination. Mutations in *SOX2* can lead to increased proliferation rates and reduced cell death [284]. *SOX2* also regulates the Notch signalling pathway, which controls keratinocyte differentiation and the skin’s barrier function [285,286]. Thus, mutations in *NOTCH1* can also be detected in SCC. Other genes involved in the pathogenesis of some SCC cases include *NFE2L2*, which regulates the response to oxidative stress and keratinocyte differentiation, and *FAT1*, which codes for desmosome proteins such as DSG1–4 [269,270]. The standard treatment for SCC is surgical excision, aiming to remove the tumour and surrounding affected tissue completely. Targeted gene therapy for SCC has yet to be developed [287].

## 4. Conclusions

The development of the skin during embryogenesis is tightly regulated by various molecular mechanisms that coordinate to ensure its proper functioning. Despite advanced technological methods offering comprehensive information on the skin’s structure and components, the molecular pathways orchestrating its formation still need further investigation. An in-depth examination of the heterogeneity of cell populations within the developing skin tissues could provide unprecedented insights into lineage specification and intercellular signalling dynamics during embryogenesis. The potential role of non-coding RNAs, cellular crosstalk, and epigenetic regulation have also been considered. Many inherited skin diseases and epidermal-derived cancers result directly from mutations in various molecular pathways. Therefore, as discussed in this review, a comprehensive understanding of the genes implicated in skin ontogeny and their relationship to epidermal pathology could enable researchers to develop laboratory-based manipulations to treat skin diseases. However, the practical application of such approaches is still in its early stages, with only a few genetic and molecular therapies currently in clinical trials.

## Figures and Tables

**Table 1:** Genes implicated in congenital skin disorders and their chromosomal loci.

Skin Disease	Gene	Chromosomal Locus	Ref.
Nevoid basal cell carcinoma syndrome	*PTCH1*	9q22.32	[151,200]
Epidermolysis bullosa simplex	*KRT5*	12q13.13	[164]
	*KRT14*	17q21.2	[164]
	*DST*	6p12.1	[161]
	*PLEC*	8q24.3	[162]
	*EXPH5*	11q22.3	[163]
	*KLHL24*	3q27.1	[166]
	*CD151*	11p15.5	[168]
Junctional epidermolysis bullosa	*LAMA3*	18q11.2	[164]
	*LAMB3*	1q32.2	[164]
	*LAMC2*	1q25.3	[164]
	*ITGA6*	2q31.1	[171]
	*ITGB4*	17q25.1	[171,173]
	*ITGA3*	17q21.33	[175,201]
	*COL17A1*	10q25.1	[178,181]
Dystrophic epidermolysis bullosa	*COL7A1*	3p21.31	[185]
Kindler epidermolysis bullosa	*FERMT1*	20p12.3	[193,194]
Hypohidrotic ectodermal dysplasia	*EDA*	Xq13.1	[202]
	*IKBKG*	Xq28	[203]
	*WNT10A*	2q35	[204]
	*EDAR*	2q13	[204]
	*EDARADD*	1q42.3-q43	[204]

Data are retrieved from “The Human Protein Atlas” [205]. CD151: Cluster of differentiation 151. COL17A1: Collagen type XVII alpha 1 chain. COL7A1: Collagen type VII alpha 1 chain. DST: Dystonin. EDA: Ectodysplasin A. EDAR: Ectodysplasin A receptor. EDARADD: EDAR-associated death domain. EXPH5: Exiphilin 5. FERMT1: FERM domain containing kindlin 1. IKBKG: Inhibitor of nuclear factor kappa B kinase regulatory subunit gamma. ITGA3: Integrin subunit alpha 3. ITGA6: Integrin subunit alpha 6. ITGB4: Integrin subunit beta 4. KLHL24: Kelch-like member 24. KRT14: Keratin 14. KRT5: Keratin 5. LAMA3: Laminin subunit alpha 3. LAMB3: Laminin subunit beta 3. LAMC2: Laminin subunit gamma 3. PLEC: Plectin. PTCH1: Patched 1.

**Table 2:** Genes implicated in skin cancers and their chromosomal loci.

Skin Cancer	Gene	Chromosomal Locus	Ref.
Basal cell carcinoma	*PTCH1*	9q22.32	[240]
	*PTCH2*	1p34.1	[243]
	*SMO*	7q32.1	[243]
	*SUFU*	10q24.32	[243]
	*TP53*	17p13.1	[245]
	*MYCN*	2p24.3	[243]
	*TERT*	5p15.33	[257]
	*DPH3*	3p25.1	[258]
Squamous cell carcinoma	*TP53*	17p13.1	[260,261]
	*CDKN2A*	9p21.3	[262]
	*CCND1*	11q13.3	[263]
	*MYC*	8q24.21	[263,264]
	*FGFR1*	8p11.23	[265]
	*FGFR2*	10q26.13	[265]
	*FGFR3*	4p16.3	[265]
	*EGFR*	7p11.2	[266]
	*TP63*	3q28	[48]
	*SOX2*	3q26.33	[267]
	*NOTCH1*	9q34.3	[268]
	*NFE2L2*	2q31.2	[269]
	*FAT1*	4q35.2	[270]

Data are retrieved from “The Human Protein Atlas” [205]. CCND1: Cyclin D1. CDKN2A: Cyclin-dependent kinase inhibitor 2A. DPH3: Diphthamide biosynthesis 3. EGFR: Epidermal growth factor receptor. FAT1: FAT atypical cadherin 1. FGFR1: Fibroblast growth factor receptor 1. FGFR2: Fibroblast growth factor receptor 2. FGFR3: Fibroblast growth factor receptor 3. MYC: MYC proto-oncogene, bHLH transcription factor. MYCN: MYCN proto-oncogene. NFE2L2: Nuclear factor-erythroid 2-like bZIP transcription factor 2. NOTCH1: Neurogenic locus Notch homolog protein 1. PTCH1: Patched 1. PTCH2: Patched 2. SMO: Smoothened, frizzled class receptor. SOX2: SRY-box transcription factor 2. SUFU: Suppressor of fused homolog. TERT: Telomerase reverse transcriptase. TP53: Tumour protein p53. TP63: Tumour protein p63.

## Abbreviations

AP-2	Activating protein 2
BCC	Basal cell carcinoma
BMP	Bone morphogenetic protein
Ca^2+^	Calcium ion
CAS9	CRISPR-associated protein 9
CCND1	Cyclin D1
CD151	Cluster of differentiation 151
CDH1	Cadherin 1
CDKN2A	Cyclin-dependent kinase inhibitor 2A
cDNA	Complementary deoxyribonucleic acid
CLDN23	Claudin 23
CLDN6	Claudin 6
COL17A1	Collagen type XVII alpha 1 chain
COL7A1	Collagen type VII alpha 1 chain
CRISPR	Clustered regularly interspaced short palindromic repeats
CSF1R	Colony-stimulating factor 1 receptor
DCs	Dermal condensates
DEB	Dystrophic epidermolysis bullosa
DKK	Dickkopf WNT signalling pathway inhibitor
DPH3	Diphthamide biosynthesis 3
DSG	Desmoglein
DST	Dystonin
E	Embryonic day
EB	Epidermolysis bullosa
EBS	Epidermolysis bullosa simplex
EDA-A1	Ectodysplasin-A1
EDAR	Ectodysplasin A receptor
EDARADD	EDAR-associated death domain
EDI200	Ectodysplasin-A1 replacement therapy
EDNRB	Endothelin receptor type B
EGFR	Epidermal growth factor receptor
EN1	Engrailed homeobox 1
EXPH5	Exiphilin 5
FAT1	FAT atypical cadherin 1
FBXW7	F-box and WD repeat domain containing 7
FERMT1	FERM domain containing kindlin 1
FGF	Fibroblast growth factor
FGFR	Fibroblast growth factor receptor
FOXA1	Forkhead box protein A1
FOXD3	Forkhead box D3
FOXI1	Forkhead box I1
GLI	Glioma-associated oncogene
GPCR	G protein-coupled receptor-like protein
GRHL3	Grainyhead-like transcription factor 3
HED	Hypohidrotic ectodermal dysplasia
HES1	Hes family bHLH transcription factor 1
HF	Hair follicle
HH	Hedgehog
HPV	Human papillomavirus
ID2	Inhibitor of DNA binding 2
IKBKG	Inhibitor of nuclear factor kappa B kinase regulatory subunit gamma
IL-34	Interleukin 34
iPSCs	Induced pluripotent stem cells
IRF6	Interferon regulatory factor 6
ITGA3	Integrin subunit alpha 3
ITGA6	Integrin subunit alpha 6
ITGB4	Integrin subunit beta 4
JEB	Junctional epidermolysis bullosa
KIT	KIT proto-oncogene
KLF4	Krüppel-like factor 4
KLHL24	Kelch-like member 24
KRT	Keratin
KS	Kindler syndrome
LAMA3	Laminin subunit alpha 3
LAMB3	Laminin subunit beta 3
LAMC3	Laminin subunit gamma 3
LEF1	Lymphoid enhancer binding factor 1
MAPK	Mitogen-activated protein kinases
MITF	Melanocyte-inducing transcription factor
MYC	MYC proto-oncogene, bHLH transcription factor
MYCN	MYCN proto-oncogene
MYH14	Myosin heavy chain 14
NBCCS	Nevoid basal cell carcinoma syndrome
NF-κB	Nuclear factor kappa B
NFE2L2	Nuclear factor-erythroid 2-like bZIP transcription factor 2
NICD	Notch intracellular domain
NOTCH1	Neurogenic locus Notch homolog protein 1
OVOL1	Ovo like transcriptional repressor 1
P	Postnatal day
PAX3	Paired box 3
PDGF	Platelet-derived growth factor
PDGFRA	Platelet-derived growth factor receptor alpha
PERP	P53 apoptosis effector related to PMP22
PI3K	Phosphatidylinositol-3-kinase
PKC	Protein kinase C
PLEC	Plectin
PTCH1	Patched 1
PTCH2	Patched 2
Rb	Retinoblastoma
RBPJ	Recombination signal binding protein for immunoglobulin kappa J region
RUNX	RUNX family transcription factor
SCC	Squamous cell carcinoma
scRNA-seq	Small conditional ribonucleic acid sequencing
SDC1	Syndecan 1
SFN	Stratifin
SHH	Sonic Hedgehog
SMO	Smoothened, frizzled class receptor
SOX	SRY-box transcription factor
SUFU	Suppressor of fused homolog
TALEN	Transcription activator-like effector nuclease
TAZ	Transcriptional coactivator with PDZ-binding motif
TERT	Telomerase reverse transcriptase
TGF	Transforming growth factor
TNF	Tumour necrosis factor
TP53	Tumour protein p53
TP63	Tumour protein p63
YAP	Yes1-associated transcriptional regulator
ZNF750	Zinc finger protein 750
